# Delimiting cryptic pathogen species causing apple Valsa canker with multilocus data

**DOI:** 10.1002/ece3.1030

**Published:** 2014-03-19

**Authors:** Xuli Wang, Rui Zang, Zhiyuan Yin, Zhensheng Kang, Lili Huang

**Affiliations:** 1 State Key Laboratory of Crop Stress Biology for Arid Areas, College of Plant Protection, Northwest A&F UniversityYangling, 712100, China; 2 State Key Laboratory for Biology of Plant Diseases and Insect Pests, Institute of Plant Protection, Chinese Academy of Agricultural SciencesBeijing, 100193, China

**Keywords:** Valsa, species delimitation, host-range expansion, molecular dating, ancestral host reconstruction

## Abstract

Fungal diseases are posing tremendous threats to global economy and food safety. Among them, Valsa canker, caused by fungi of *Valsa* and their *Cytospora* anamorphs, has been a serious threat to fruit and forest trees and is one of the most destructive diseases of apple in East Asia, particularly. Accurate and robust delimitation of pathogen species is not only essential for the development of effective disease control programs, but also will advance our understanding of the emergence of plant diseases. However, species delimitation is especially difficult in *Valsa* because of the high variability of morphological traits and in many cases the lack of the teleomorph. In this study, we delimitated species boundary for pathogens causing apple Valsa canker with a multifaceted approach. Based on three independent loci, the internal transcribed spacer (ITS), *β*-tubulin (Btu), and translation elongation factor-1 alpha (EF1*α*), we inferred gene trees with both maximum likelihood and Bayesian methods, estimated species tree with Bayesian multispecies coalescent approaches, and validated species tree with Bayesian species delimitation. Through divergence time estimation and ancestral host reconstruction, we tested the possible underlying mechanisms for fungal speciation and host-range change. Our results proved that two varieties of the former morphological species *V. mali* represented two distinct species, *V. mali* and *V. pyri*, which diverged about 5 million years ago, much later than the divergence of their preferred hosts, excluding a scenario of fungi–host co-speciation. The marked different thermal preferences and contrasting pathogenicity in cross-inoculation suggest ecological divergences between the two species. Apple was the most likely ancestral host for both *V. mali* and *V. pyri*. Host-range expansion led to the occurrence of *V. pyri* on both pear and apple. Our results also represent an example in which ITS data might underestimate species diversity.

## Introduction

Species are a fundamental unit of biodiversity (Hull 1977; Sites and Crandall 1997; Bacon et al. 2012), and robust species delimitations are of critical importance in many areas of biology. For fungi, delimitation of species even has tangible consequences for the society, especially for those groups causing infectious plant diseases that pose serious threat to food safety and ecosystem health (Fisher et al. 2012). There are many cases where closely related species, even morphologically indistinguishable, can differ greatly in their virulence and host specificity, and thus the level of threat they pose to agriculture and forestry. Specific and precise species delimitation of these pathogens is essential for effective disease control and quarantine regulations, and for sustainable management practices in agriculture and forestry (Rintoul et al. 2012). However, methods for species delimitation are very limited in many groups, often relying on very limited morphological and cultural characteristics that vary widely during life history and can be easily influenced by nonheritable factors such as environmental conditions and sexual forms. The invention of dual nominal system is inadequate compromise for fungi that have contrasting morphology between sexual (teleomorphic) and asexual (anamorphic) forms. Although some theoretical and methodological innovations have been achieved in recent years, species delimitation still remains a major challenge facing biodiversity conservation and disease management.

Molecular genetics has revolutionized our ability for robust species delimitation (Lumbsch and Leavitt 2011; Leavitt et al. 2012). Genetic materials, providing highly variable and stable characters, have been extensively used in fungi systematics since its introduction. With ever-increasing availability of genetic data and improvement of analytical methodologies, DNA-based approaches play increasing role in the recognition of diversity of fungi, especially in groups that would otherwise hardly be recognized using classical phenotypic characteristics (Leavitt et al. 2012 and refs therein). The discovery of cryptic diversity has been particularly prevalent in those groups. Recent biodiversity studies witnessed a surge of interest in identifying species using DNA sequence data, for example, DNA barcoding (Schoch et al. 2012). DNA barcoding, which identifies species based on single-locus genealogy and assumes the existence of “barcoding gap” between inter- and intraspecific divergences, does have facilitated biodiversity inventory, but it also becomes increasingly apparent that single-locus data represent the history of a single gene that might not be representative of organismal history (Rosenberg 2002). In addition, it is subjective to place the species boundaries in single-gene genealogies, which will create uncertainty on species' limits (Taylor et al. 2000). Especially, due to stochastic lineage sorting across genomes and ongoing gene flow during speciation, for closely related fungi, one marker is definitely not enough (Dupuis et al. 2012). As a consequence, multiple independent loci are required for reliable species delimitation (Rintoul et al. 2012). Multilocus data have been successfully applied to delimitate several closely related plant pathogenic ascomycete fungi, for example, in *Neurospora* (Dettman et al. 2003), *Fusarium* (O'Donnell et al. 2004) and *Septoria* (Verkley et al. 2013). In addition, using recently developed theoretical models that combine species phylogenies and gene genealogies via ancestral coalescent processes, multilocus sequence data can provide support for different species delimitations (Yang and Rannala 2010).

**Figure 1:**
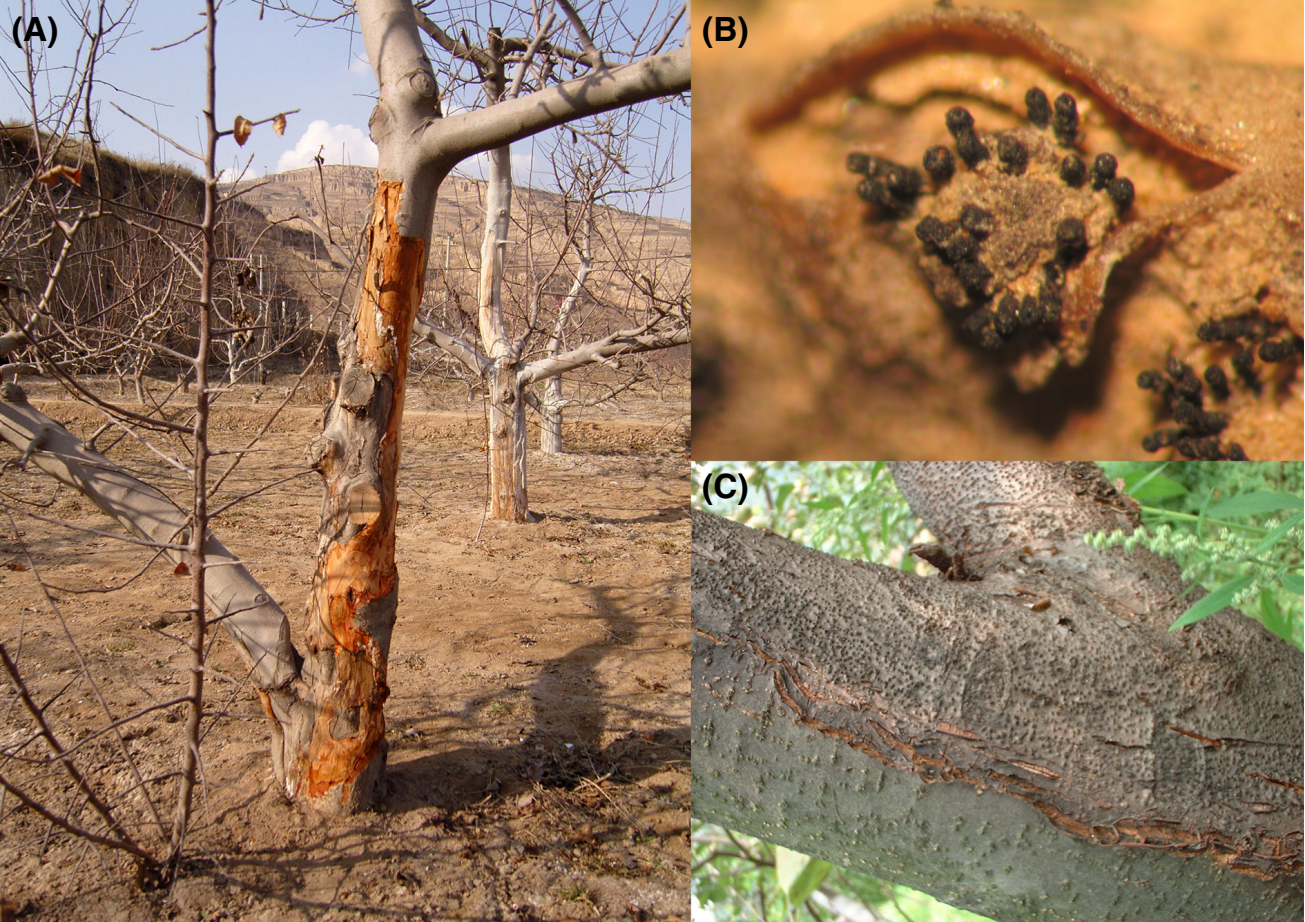
Infection of apple (*Malus sp*.) by *Valsa mali* (A) with conidiomata (C) and, in rare occasions, the ascostromata (B) formed in the canker.

Species of *Valsa* and their *Cytospora* anamorphs affect more than 70 species of woody shrubs and trees and cause serious threats to fruit and forest trees through the perennial canker worldwide (Agrios 1997). In particular, valsa canker is one of the most destructive diseases of apple (*Malus* sp.) in East Asia (Chen et al. 1987; Uhm and Sohn 1995; Wang et al. 2005; Lee et al. 2006; Abe et al. 2007), seriously impacting tree productivity through developing cankers on trunks and scaffold limbs or causing diebacks of twigs (Fig. 1). As the pathogen penetrates extensively into the host phloem and xylem (Tamura and Saito 1982), this disease cannot be controlled effectively through chemical treatments (Abe et al. 2007). It is pivotal to accurately delimitate pathogen species for the development of effective disease control methods and sustainable management strategies. However, species delimitation is notoriously difficult in *Valsa* because of the high variability of morphological traits and in many cases the lack of the teleomorph (Adams et al. 2002). This is exemplified by a hundred years of debates about the association of pathogens causing Valsa canker on apple in East Asia (see Wang et al. 2011 for historical review therein).

Our earlier studies of isolates from cankered apple trees based on the internal transcribed spacers and 5.8S region (ITS) of the nuclear ribosomal repeat unit revealed two lineages within isolates of *V. mali*. Due to failing to find stable morphological differences and considering high stochastic nature of singe-locus genealogy, we putatively recognized these two lineages as varieties, although some differences in pathogenicity and cultural characteristics have been observed in experimental conditions (Wang et al. 2011). However, given the seven fixed differences observed in ITS sequences between two lineages (suggesting cessation of gene flow for prolonged times) and differential pathogenicity manifested on apple and pear (implying host specificity), it is reasonable to hypothesize that these two lineages might represent distinct evolutionary units, that is, species.

So the goal of this study is to test this hypothesis from multilocus perspective and delimitation species boundary for pathogens causing apple Valsa canker with a multifaceted approach, integrating evidence from multilocus phylogenetics, Bayesian species delimitation, and phenotypes (cultural characteristics, thermal preferences, and pathogenicity) under experimental conditions. Specifically, based on three independent nuclear loci, the internal transcribed spacer (ITS), *β*-tubulin (Btu), and translation elongation factor-1 alpha (EF1*α*), we (1) test whether two lineages recognized with earlier ITS sequence data are supported by other genomic loci; (2) delimitate species by integrating all evidences we have obtained; (3) infer the evolutionary scenario giving rise to current fungi–host relationship; and (4) evaluate the performance of ITS sequence for *Valsa* diversity estimation.

## Materials and Methods

### Pathogen sampling, DNA extraction, amplification, and sequencing

Pathogen sampling and isolation, DNA extraction, and amplification and sequencing ITS have been detailed in Wang et al. (2011) and Zang et al. (2012). A total of 150 single-spore isolates were obtained from apple (*Malus* sp.), and additional nine isolates were obtained from pear (*Pyrus* sp.). Based on ITS sequence data, 145 isolates from apple were recognized as *V. mali* var. *mali*, and the remained five isolates and all nine isolates from pear were *V. mali* var. *pyri*. All herbarium specimens and isolates studied were deposited at the College of Plant Protection, Northwest A&F University, and State Key Laboratory of Crop Stress Biology for Arid Areas, China. In this study, a subsample that included 20 isolates from apple, of which 16 isolates were *V. mali* var. *mali* and four isolates, were *V. mali* var. *pyri*, and five isolates (all were *V. mali* var. *pyri*) from pear were subjected to analyses of two additional loci, translation elongation factor-1 alpha (EF1*α*), and the *β*-tubulin (Btu). We also sequenced five isolates of *V. malicola* as close outgroups and one isolate of *V. leucostoma* (together with two sequences from GenBank) as more distant outgroups. The partial sequence of EF1*α* gene was amplified with primers EF1-728F (5′- CAT CGA GAA GTT CGA GAA GG-3′) and EF1-986R (5′-TAC TTG AAG GAA CCC TTA CC-3′) (Carbone et al. 1999). A partial Btu gene sequence was amplified using primers Bt2a (5′-GGT AAC CAA ATC GGT GCT GCT TTC-3′) and Bt2b (5′-ACC CTC AGT GTA GTG ACC CTT GGC-3′) (Glass and Donaldson 1995). All reactions were carried out in 25 mL volumes composed of 1X reaction buffer, 1.5 mmol/L MgCl_2_, 2.5 mmol/L of each dNTPs, 5 pmol/L each primer, 0.6 unit Taq DNA polymerase (Promega, Shanghai, China), and 30–50 ng genomic DNA. The following thermal profile for PCR was applied: An initial denaturation at 94°C for 4 min was followed by 38 cycles of 30 s at 94°C, 30 s at 55°C (EF1*α*) or 61°C (Btu) and 30 s at 72°C, and a final extension at 72°C for 5 min. The PCR products were purified with the QIAquickH PCR Purification Kit (QIAGEN, Valencia, CA) and directly sequenced with the ABI PRISM BigDye Terminator V3.1 Cycle Sequencing Kit (Applied Biosystems, Foster City, CA). The sequences were resolved on an ABI 3130XL automated sequencer. All sequences have been deposited in GenBank under accession numbers listed in Table S1.

### Sequence analyses

All raw sequences were checked with Sequencing Analysis 5.2 (Applied Biosystems), and the unresolved sites of both ends were removed. The trimmed sequences were aligned using Clustal X 1.83 (Thompson et al. 1997) and further inspected manually. Summary statistics, including gene diversity (Hd) and nucleotide diversity (*π*), were determined using DnaSP version 5.10.01 (Librado and Rozas 2009). Genetic distances between groups were calculated with MEGA 5.05 (Tamura et al. 2011). Rough relationships between unique sequences were inspected through media-joining networks (Bandelt et al. 1999) using the Network 4.6 (http://www.fluxus-engineering.com/). A NeighborNet network based on p-distance was constructed for the concatenated alignment using SplitsTree4 version 4.12.3 (Huson and Bryant 2006).

### Phylogenetic analyses and molecular dating

The appropriate models of DNA evolution for the data were inferred using ModelTest 3.7 program (Posada and Crandall 1998). According to the Akaike information criterion, the best-fit model is TrNef+I for ITS, K81uf+I for EF1*α,* and HKY+I for Btu. A TIMef+I model was selected for the concatenated sequences of three loci. Phylogenetic analyses were performed using both maximum likelihood (ML) and Bayesian methods for each singe-locus data and for concatenated data. No significant conflict has been detected between three loci in incongruence length difference test (Huelsenbeck et al. 1996; ITS vs. Btu, *P *=* *1.00; ITS vs. Ef1*α*,*P *=* *1.00; Btu vs. Ef1*α*,*P *=* *0.11). We also run the Bayesian analysis under partitions with each locus assigned a specific best-fit substitution model selected by ModelTest. ML analysis was carried out using PHYML 3.0 (Guindon and Gascuel 2003). Topological robustness was assessed through 1000 bootstrapping replicates. Bayesian analysis was conducted with MrBayes version 3.1.2 (Ronquist and Huelsenbeck 2003). Analyses were initiated with random starting trees. Each analysis ran for 10^6^ generations with four Markov chains employed. Trees were sampled every 100 generations, and the “temperature” parameter was set to 0.2. The first 25% trees were discarded as burn-in after a careful inspection of the stationary state and the convergence of the chains with TRACER 1.5 (Rambaut and Drummond 2007).

To get insights into the temporal scale for speciation, the divergence times were estimated using BEAST 1.7.4 (Drummond and Rambaut 2007) with a relaxed molecular clock approach, as the molecular clock model was rejected by likelihood ratio tests for all single-gene data and the concatenated data (*P *<* *0.0001, Table S2). The rate change was explicitly modeled using uncorrelated lognormal distribution across trees. We ran the analyses with three different tree prior, one speciation prior, a Yule process (pure birth process), and two coalescent prior, a constant size and an extended Bayesian skyline. Tree topology and age were simultaneously estimated with branch length and substitution model parameters. Two million Markov chain Monte Carlo (MCMC) searches were performed and sampled every 2000 generations. Convergence of the MCMC chains was checked with TRACER 1.5 (Rambaut and Drummond 2007). Maximum clade credibility (MCC) tree, posteriors, means, and 95% highest posterior densities (HPDs) for ages of nodes were indentified and annotated using TreeAnnotator 1.6.2 (Drummond and Rambaut 2007). In the absence of relevant fossil and other evidence for calibration, we used the substitution rates reported for the ITS sequences (2.52 × 10^−9^ per site per year) (Takamatsu and Matsuda 2004) to estimate the time to the most recent common ancestor for all clades. This substitution rate for ITS sequences is comparable with that reported in other group of Ascomycota, for example, 2.43 × 10^−9^ for Parmeliaceae (Leavitt et al. 2012) and 1.46 ± 1.3 × 10^−9^ for Eurotiomycete (Kasuga et al. 2002).

### Species tree estimation and validation

Establishing a preliminary hypothesis of species boundaries using individual locus and concatenated sequence data provides a reasonable starting point for species delimitation. However, inferring a species tree using these results has been shown to be misleading under certain divergence scenarios (Degnan and Rosenberg 2006, 2009; Leaché 2009; Leavitt et al. 2012). We employed a Bayesian Markov Chain Monte Carlo (MCMC) method (^∗^BEAST) to jointly estimate multiple gene trees embedded in a shared species tree under the multispecies coalescent (Heled and Drummond 2010). ^∗^BEAST estimates the species tree that is most probably given the multiindividual multilocus sequence data and incorporates uncertainty associated with the coalescent process and gene genealogies. This method is considerably more accurate than supermatrix approaches (Heled and Drummond 2010). We conducted ^∗^BEAST analyses on all three datasets with species defined a priori according to the results of phylogenetic analyses. Analyses were partitioned by locus, and all parameters were unlinked across loci with an uncorrelated lognormal model of rate variation assumed for each locus. A Yule process prior was used for species tree, and the population size model was set to piecewise linear and constant root. Three independent runs were executed in BEAST version 1.7.4 (Drummond and Rambaut 2007) with each run consisting of 100 million generations sampling every 5000 generations. Convergence of parameter values was assessed by plotting the marginal probabilities using the program TRACER version 1.5 (Rambaut and Drummond 2007).

We used the program Bayesian Phylogenetics and Phylogeography (BPP v2.2) (Yang and Rannala 2010) to delimit species using the reversible-jump Markov chain Monte Carlo (rjMCMC) method. This method infers the joint posterior distribution of species delimitation and species tree and yields a posterior probability associated with the existence of each species. Using all the three loci, we infer the joint posterior distribution for the hypothetical guide tree. The species tree estimated by *BEAST was used as guide tree. The program assesses the probability of the node separating the two species exist under a general lineage species concept while accounting for lineage sorting due to time since divergence and population size. We used algorithm 0 with and parameterized both ancestral population size (*Θ*) and root age (*τ*_o_) using a gamma distribution, *Γ* (2, 1000). Three independent analyses were run with different starting seeds for 100,000 generations with a burn-in of 20,000 and thinning every five generations.

The taxonomic distinctiveness of delimited species was also assessed using the genealogical sorting index (*gsi*; Cummings et al. 2008), a quantitative measure of the degree to which ancestry of delimited species is exclusive. *gsi* is on a scale from 0 to 1, where 1 indicates complete monophyly. We calculated *gsi* for each delimited species from each locus using the Genealogical Sorting Index web server (http://www.genealogicalsorting.org). The 50% majority-rule consensus gene trees inferred by MrBayes were used as input trees. The null hypothesis that the degree of exclusive ancestry is observed by chance alone (i.e., no divergence) was evaluated by estimating a *P* value using 10,000 permutations.

### Ancestral host reconstruction

The historical host of the hypothetical ancestors (internal nodes) was reconstructed using Bayes-DIVA implemented in RASP 2.0 Beta (Yu et al. 2010). Bayes-DIVA determines the probability of ancestral state for each node averaged over all sampled trees derived from MCMC and thus accounts for phylogenetic uncertainty in inference (Nylander et al. 2008). We sampled 10,000 trees from MCMC in *BEAST with concatenated data and generated maximum clade credibility tree from the sampled trees using TreeAnnotator 1.6.2 (Drummond and Rambaut 2007). These trees were used as inputs for ancestral state reconstruction. The analysis was run for 50,000 cycles using 10 chains. Hosts for each isolate were recorded during collection. Three kinds of hosts (including for outgroups) were observed: (1) apple, *Malus* sp., (2) pear, *Pyri* sp., and (3) peach, *Prunus* sp. The maximum number of areas in ancestral ranges was constrained to three.

## Results

### Sequence variability and genetic diversity

**Figure 2:**
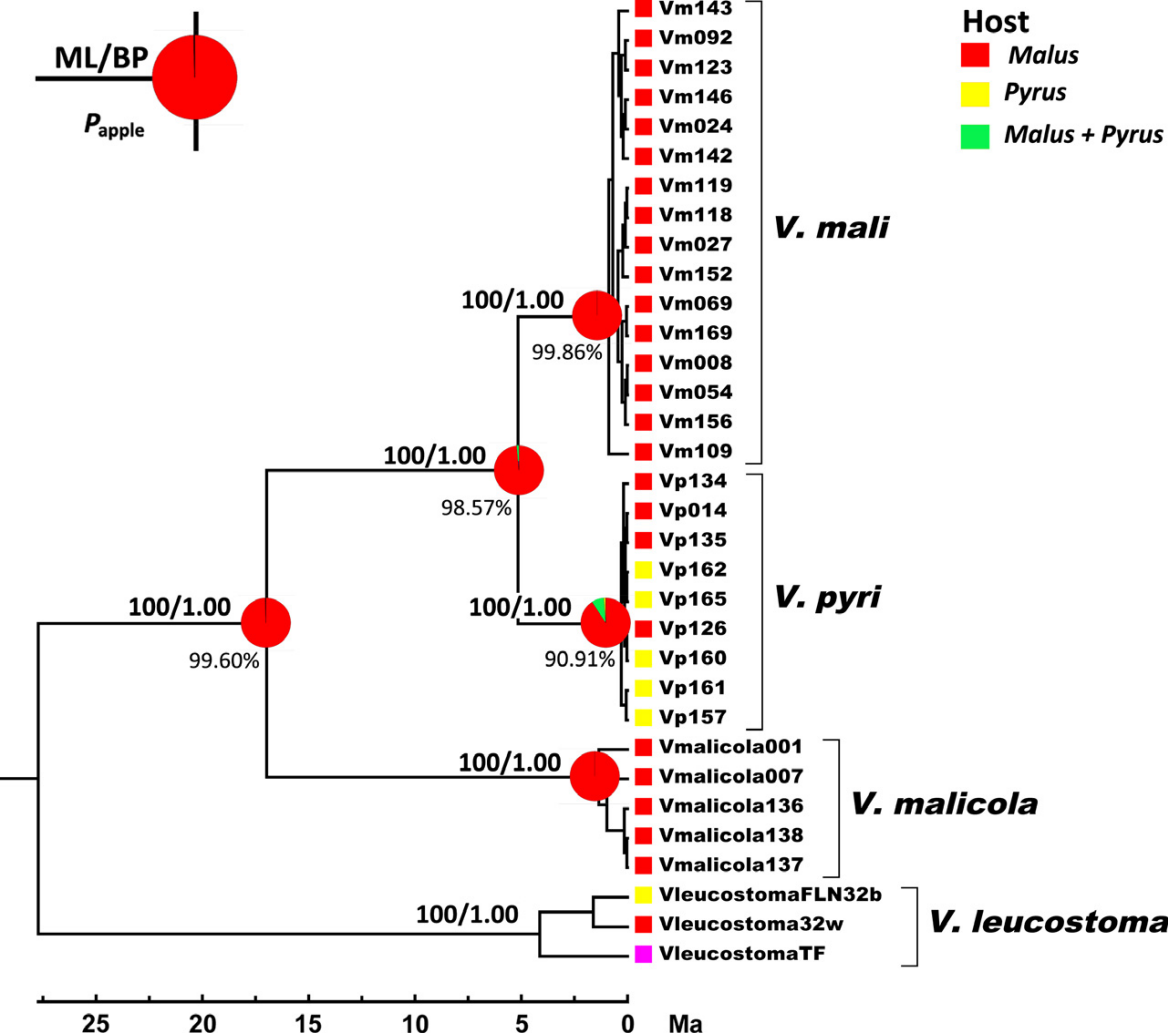
Time-calibrated phylogeny and reconstructed ancestral hosts. Shown here is the maximum clade credibility tree inferred from concatenated data using the Bayesian relaxed phylogenetic approach implemented in BEAST. Shown above the branches are bootstrap support values (%) from 1000 replicates (maximum likelihood analysis)/posterior probabilities (Bayesian inference). Ancestral hosts reconstructed from Bayes-DIVA analysis were mapped on the nodes as colored pie charts, with the relevant colors proportional to the mean probability of the ancestral host. The mean probability of the ancestral host being apple, *P*_apple_, was shown under by the nodes. Host states color codes: red, apple (*Malus*); yellow, pear (*Pyrus*); and green, both apple and pear (*Malus *+ *Pyrus*).

**Figure 3:**
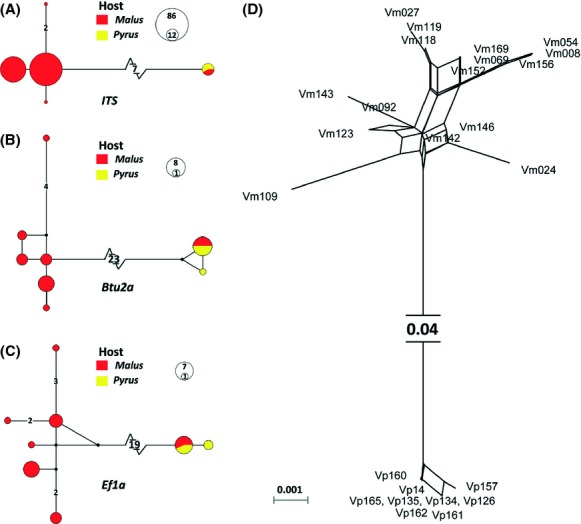
Genetic divergences between *V. mali* and *V. pyri* were shown by media-joining networks for each locus (A–C),and by NeighborNet based on p-distances for the concatenated data (D). In (A–C) branch lengths are proportional to the number of substitutions occurred except for the long branches linking haplotypes of *V. mali* and *V. pyri*, which were shortened and given in numbers. Circle sizes are proportional to the number of isolates sharing the haplotypes. Isolates from apple were depicted in red and from pear in yellow. Note: For ITS, network (A) was constructed based on 150 isolates from apple and nine isolates from pear.

The sequences variability and genetic diversity were summarized in Table 1. For ITS, we reported statistics basing on the subsampled 30 isolates (which have been sequenced for EF1*α* and Btu in this study) in order to make cross-locus comparisons more meaningful. In general, EF1*α* and Btu are much more polymorphic than ITS sequences, with 26.0% (77/296) and 16.4% (84/512) of sites been variable, six or four times more than that of ITS (3.91%, 23/588). The mean genetic distances (p-distance) between *V. mali* and *V. pyri* (recognized in this study) are 5% for Btu and 8% for EF1*α*, more than fourfold for ITS (1.2%). The genetic distances between *V. mali* and *V. malicola* are similar to that between *V. pyri* and *V. malicola*, with 13% for Btu and 25% for EF1*α*. Similarly, there are much more intraspecific variations in EF1*α* and Btu than in ITS (Table 2). Gene diversity and nucleotide diversity are comparable in *V. mali* and *V. pyri,* while *V. malicola* harbors slightly more diversity in EF1*α* and Btu. The mutation that occurs between unique sequences for each locus is shown by network in Fig. 3A–C. The NeighborNet network (Fig. 3D) shows that there is essentially no reticulation between two groups of isolates in the former *V. mali*, which correspond to the two subclades revealed in later phylogenetic analyses (Fig. 2).

**Table 1:** Estimates of genetic distances between species. Means and standard errors of the number of base differences per site (p-distance) from averaging over all sequence pairs between groups are shown. Standard errors were obtained by 1000 bootstrapping replicates. Ambiguous sites were removed before distance calculation

Locus	Length	S	*V. mali* vs. *V. pyri*	*V. mali* vs. *V. malicola*	*V. pyri* vs. *V. malicola*
ITS	588 bp	23	0.012 ± 0.004	0.033 ± 0.007	0.035 ± 0.007
Btu	512 bp	84	0.051 ± 0.009	0.132 ± 0.014	0.130 ± 0.015
EF1*α*	296 bp	77	0.080 ± 0.015	0.252 ± 0.026	0.252 ± 0.025

S, number of segregating sites.

**Table 2:** Summary of genetic statistics of three loci (in the order of ITS/Btu/EF1*α*) for three Valsa species on apple

Species	*N*	*S*	Hd	*π*
*V. mali*	16/16/16	1/8/8	0.233/0.817/0.767	0.0004/0.0039/0.0092
*V. pyri*	9/9/9	0/1/1	0/0.222/0.389	0/0.0005/0.0014
*V. malicola*	5/5/5	0/10/8	0/0.700/0.900	0/0.0089/0.0148

*N*, number of isolates; *S*, number of segregating sites; Hd, gene diversity; *π*, nucleotide diversity.

### Phylogenetic inferences and species tree estimation

In all single-gene analyses, ML and the Bayesian inferences obtained largely congruent results differing only in arrangements within major clades (Fig. S1). Phylogenetic analyses of the concatenated alignment with single overall model and with each locus assigned a specific best-fit substitution model obtained the identical tree topology. These tree topologies are identical to that of MCC tree obtained in BEAST (Fig. 2). In general, all phylogenetic analyses revealed two reciprocal monophyletic clades within the former morphological species *V. mali*, which we have found in our early study with only ITS sequences (Wang et al. 2011). These clades were strongly supported in the concatenated analysis by bootstrap values and Bayesian posterior probabilities of 100/1.00. We assigned these two clades *V. mali* and *V. pyri*, respectively (see Discussion).

**Figure 4:**
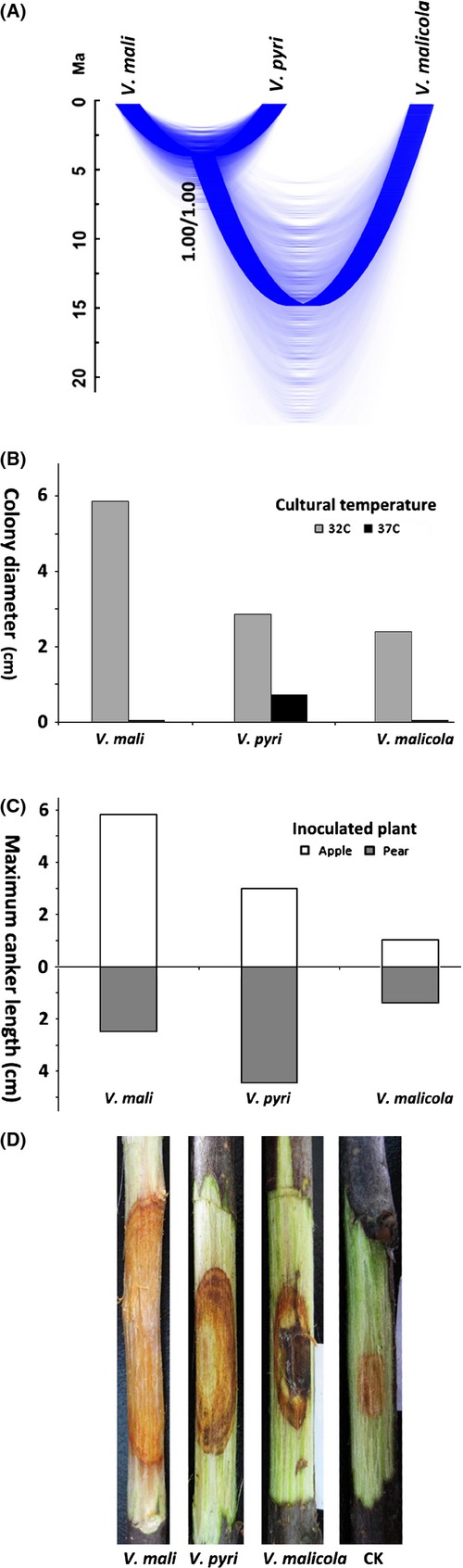
Summary of evidences supporting two distinct species *V. mali* and *V. pyri*. (A) Species tree jointly estimated using ^∗^BEAST. The consensus tree was overlaid on postburnin trees sampled in MCMC analysis. The time scale is in unit of million years (Ma). (B) Histograms show colony diameters for the isolates of three species cultured on PDA medium for 3 days at 32°C and 37°C. (C) Bar charts show the maximum canker length (cm) developed by *V. mali*,*V. pyri,* and *V. malicola* after 10 days of inoculation during cross-pathogencity test. (D) Pictures of fungal infection on 2-year-old apple twigs during artificial inoculation. Clear difference in canker sizes was observed between *V. mali* and *V. pyri*. Data for generating (B and C) are from Wang et al. (2011).

The species trees estimated by ^∗^BEAST are presented in Figure 4A. Speciation between *V. mali* and *V. pyri* is strongly supported (speciation probabilities 1.00). This species tree is also strongly supported by Bayesian species delimitation in BPP. Values of *gsi* indicate a high degree of exclusive ancestry within delimited species for the all loci (Table 3).

**Table 3:** Genealogical sorting index (*gsi*) of three delimited Valsa species for gene trees. All *P* values based on 10,000 permutations are <0.0001

Locus	ITS	Btu	EF1*α*	All combined
*V. mali*	0.87	1.00	1.00	1.00
*V. pyri*	0.85	0.85	0.85	0.85
*V. malicola*	1.00	1.00	1.00	1.00

### Divergence times and ancestral hosts

Divergence times for major nodes, estimated with Bayesian relaxed clock method, were summarized in Table 4 and also shown in Figure 2. Running BEAST with different tree priors obtained similar results, with very close means and overlapping 95% HPDs. Both *V. mali* and *V. pyri* coalescented very recently, with mean dates less than one million years ago (Ma). Divergence between *V. mali* and *V. pyri* occurred around 5 Ma (95% HPDs, 2.46–8.14 Ma with different tree priors), and the common ancestor of *V. mali* and *V. pyri* diverged with *V. malicola* about 15–18 Ma (95% HPDs, 7.13–26.54).

**Table 4:** Means and 95% HPDs (in brackets) for divergence dates estimates using different tree priors with relaxed molecular clock method implemented in BEAST1.7.4

MRCA	Coalescent: constant size	Coalescent: EBSP	Speciation: Yule process
*V. mali*	0.89 [0.38, 1.45]	0.78 [0.32, 1.32]	1.08 [0.47, 1.80]
*V. pyri*	0.30 [0.08, 0.56]	0.21 [0.04, 0.42]	0.43 [0.11, 0.82]
(*V. mali*,*V. pyri*)	5.16 [2.78, 7.79]	5.27 [2.86, 8.14]	5.12 [2.46, 7.94]
(*V. mali*,*V. malicola*)	16.98 [9.97, 24.86]	17.92 [10.50, 26.54]	14.77 [7.13, 22.93]

MRCA, most recent common ancestor; EBSP, extended Bayesian skyline plot. Dates are given in million years ago.

The reconstructed historical hosts of the hypothetical ancestors were depicted with pie charts on the respective nodes in the MCC tree (Fig. 2). As indicated by the colored pie charts in Figure 2, Bayes-DIVA analysis indicated that apple (*Malus*) was the ancestral host for *V. mali* (*P *=* *99.86%), and the ancestral host of *V. pyri* was most probably apple (*P *=* *90.91%). Apple was also the ancestral host for the common ancestor of *V. malicola* and *V. mali* (*P *=* *99.60%).

## Discussions

### Cryptic species and host-range expansion

Fungal diseases evoke tremendous threats on a wide range of host plants at an increasing rate (Jones et al. 2008). Specific and precise delimitation of fungal species is not only essential for the development of effective disease control program, but also will advance our understanding of the emergence of plant diseases. However, species delimitation in fungi still remains a challenging task. This is in part due to the paucity of reliable morphological characters in some groups, but lacking of consensus on a definition of the term “species” aggravates the situation. With theoretical and methodological advances, most biologists agree that the “species phenomenon” is real, which is expressed by consistent discontinuities along morphological, genetic, and/or ecological axes (Dobzhansky 1937; Sterelny 1999; Coyne and Orr 2004; Hausdorf 2011).

Our multilocus phylogenetic analyses consistently revealed two strongly supported monophyletic clades with the former morphologically indistinguishable species *V. mali* (Fig. 2). These monophyletic clades, resulting from nearly complete lineage sorting (*gsi *≥ 0.85, Table 3) in all three loci, imply cessation of gene flow for very prolonged time period and thus represent a clear genetic discontinuity. This genetic discontinuity is also clearly reflected in Bayesian species tree estimation, in which the common ancestral node was supported by a speciation probability of 1.00 (^∗^BEAST and BPP, Fig. 4A). Although we failed to capture any stable morphological difference, different thermal preferences and contrasting pathogenicity in cross-inoculation on apple and pear (implying hosts preferences, Wang et al. 2011) suggest the differential niche occupation between isolates belonging to these two clades. These evidences suggest a clear discontinuity along ecological axes (Fig. 4B–D). Taken all these evidences together, although being morphologically cryptic, it is reasonable and adequate to recognize the two clades (varieties in earlier study, Wang et al. 2011) as two distinct species, *V. mali* and *V. pyri*.

Genetically, there are 7, 23, and 19 fixed differences, respectively, that have been observed in ITS, Btu and EF1*α* between *V. mali* and *V. pyri* (Fig. 3). Culturing on the PDA medium, the colony color of *V. mali* changed from white at first to light brown in the later period, but that of *V. pyri* remained milk white throughout the cultural period (with one exception out of 14 isolates that changed to dark gray in the later period). On 20% ABA, many smaller conidiomata were produced by isolates of *V. mali*, while a few larger conidiomata were produced by the isolates of *V. pyri* (Wang et al. 2007, 2011). There were significant differences in the growth rate at high temperatures (Fig. 4B). Culturing on PDA at 32°C for 3 days, the colony diameter of *V. mali* was more than twice compared with *V. pyri*, while at 37°C *V. mali* ceased to grow but *V. pyri* could develop normal colonies (Wang et al. 2011). Artificial cross-inoculations on apple and pear revealed contrasting patterns of pathogenicity (Fig. 4C). *V. mali* was significantly more aggressive on apple than on pear, with canker length more than twofold on apple twigs than on pear twigs (MCL: 5.85 vs. 2.50 cm). On the contrary, *V. pyri* was consistently more pathogenic on pear than on apple, developing cankers on apple twigs only 2/3 the length of that on pear twigs (MCL: 3.02 vs. 4.41 cm). Geographic distributions of these two species need to be investigated in future. Current known occurrences of *V. mali* include North China, Japan and Russia, and *V. pyri* include China and Italy (Wang et al. 2011). In nature, *V. mali* exclusively occurs on apple, while *V. pyri* can be found both on apple and pear (Table S1).

The estimated divergence times between two species occurred around 5 million years ago (95% HPDs, 2.46–8.14; Table 4). This divergence time was much later than that of apple (*Malus*) and pear (*Pyrus*) occurring about 45–59 Ma (Lo and Donoghue 2012). This evidence disfavors the fungi–host co-speciation scenario and suggests that speciation of *V. mali* and *V. pyri* might involve other forces. The results of ancestral host reconstruction revealed that apple was the most likely host for most common ancestors both of *V. mali* and of *V. pyri* (Fig. 2). This result implies that *V. pyri* colonized pear through host-range expansion (i.e., colonization of a new host species while remaining pathogenic on the ancestral host). Unlike host shift (i.e., colonization of a new host species associated with the loss of the ability to infect the ancestral host), which involves very strong qualitative host specialization, host-range expansion requires nonobligate specialization but quantitative specialization often results in a higher performance on the new host (Lê Van et al. 2012). Our observations of cross-inoculation are consistent with this expectation. Isolates of *V. pyri* developed significant larger canker on pear (expended host) than on apple twigs (ancestral host) (MCL: 4.41 vs. 3.02 cm). Results of molecular dating and ancestral host reconstruction revealed that host-range expansion occurred very recently. Unfortunately, our current sampling design and molecular information are inadequate to infer the frequency and mechanism of host-range expansion for *V. pyri*.

### ITS barcoding underestimates *Valsa* diversity

Our results demonstrated that molecular approaches were more powerful to accurately resolve fungal lineages and species. However, our results also indicated that species delimitation using molecular data should be applied under feasible theoretical framework. There is a surge of interest in identifying species using DNA barcoding in biodiversity studies (Schoch et al. 2012). For its operational easiness, DNA barcoding will definitely help increasing our steps for biodiversity inventory clarification, but warnings and criticisms never fall behind the use of this method because of uncertainty of the existence of “barcoding gap”.

ITS regions are widely used in fungal diversity studies for several reasons including the available database in GenBank and the ease for amplification among distant fungal lineages (White et al. 1990). However, many studies have demonstrated that ITS alone is an insufficient variable to delimitate species predicted by other nuclear loci. There are some cases that ITS alone largely underestimates the species diversity (Gazis et al. 2011), especially in rapidly evolving or highly diverse genera or species complexes (Lacap et al. 2003; Hoffman and Arnold 2008), and in some cases to be unreliable for species identification (Harder et al. 2013). Although, there were some studies in which a clear “barcoding gap” was found between inter- and intraspecific genetic distances in ITS, the threshold varied widely. For example, mean interspecific distances range from 6% to 8% in Parmeliaceae, but its minimum can be as low as 1.5% (Del-Prado et al. 2010). Most species of *Tuber* showed 1–3% intraspecific ITS variability and >4% interspecific ITS sequence variation (Bonito et al. 2010). In the case of the *Tricholoma scalpturatum* complex, the mean value of intraspecific genetic distances was <0.2%, whereas interspecific divergence estimates ranged from 1.78% to 4.22% (Jargeat et al. 2010). Meta-analysis revealed that the average of intraspecific ITS variability of the kingdom Fungi is 2.51% and that it is lower in Ascomycota, 1.96% (Nilsson et al. 2008). All these examples suggest that there is no single unifying yet stringent threshold for inter- and intraspecific variability. In our case, we found an interspecific distance of 1.2% between *V. mali* and *V. pyri*. This value seems to be intraspecific comparing above examples. However, high divergence in Btu (5%) and EF1*α* (8%), results of phylogenetic analyses, Bayesian species delimitation, cross-inoculation test, and difference in high temperature tolerance clearly supported the distinctness of these two species (Fig. 4). Our results suggest that delimitating species based on ITS sequence alone may underestimate *Valsa* species diversity. We propose to use an integrated species delimitation procedure, which combines evidences from multiple locus phylogenetic analyses, cultural characteristics, and cross-host preference test, for this species rich fungal genus.

### Perspective on canker disease managements

As the causal agent of Valsa canker penetrates extensively into the plant phloem and xylem, the disease cannot be controlled effectively through chemical treatments (Abe et al. 2007). So beforehand prevention is of pivotal importance. In this study, we found two close related but unique species, *V. mali* and *V. pyri*, on apple and pear. The distinct features of these two pathogen species and their distinct host preferences decided that they should be evaluated independently in the disease assessment and quarantine and consequently should be treated with specific management practices.

There are many examples of interspecific hybridization having been reported (e.g., Brasier et al. 1999; Newcombe et al. 2000; Moon et al. 2004; Brasier and Kirk 2010; Stukenbrock et al. 2012), and hybridization represents one of the common mode of speciation in fungi (Giraud et al. 2008, 2010). Interspecific recombination can help to spread pathogenic alleles to cross species boundary and thus conferring improved virulence to less pathogenic species. So an impending concern relevant to two pathogen species is the likelihood for the occurrence of interspecific hybridization. The domestication of their hosts and current grafting practices create such opportunities (mating within the same host), which might not exist in the wild. As grafting between apple and pear is common in horticultural practice, grafting will provide hotbed for interspecific hybridization. Their co-existence in the apple will especially increase the likelihood of interspecific hybridization. Thus, it is important to monitor the occurrence of hybridization between these two species in the fields and carry out a priori pathogen examination before seedling grafting.

## Supporting Information

**Table S1.** Isolates, host origins, and GenBank accession numbers of isolates of *Valsa* species. Sequences for VleucostomaFLN32b were obtained from GenBank. **Table S2.** Results of likelihood ratio test of molecular clock in three loci (ITS, Btu, and EF1α).

Figure S1 Maximum likelihood tree inferred from ITS (left), Btu (middle), and EF1α (right). Bootstrapping values are shown together with Bayesian posterior probabilities at the node.

## References

[b1] Abe K, Kotoda N, Kato H, Soejima J (2007). Resistance sources to Valsa canker (*Valsa ceratosperma*) in a germplasm collection of diverse *Malus* species. Plant Breeding.

[b2] Adams GC, Surve-Iyer RS, Iezzoni A (2002). Ribosomal DNA sequence divergence and group I introns within the *Leucostoma* species *L. cinctum L. persoonii* and *L. parapersoonii* sp. nov., ascomycetes that cause Cytospora canker of fruit trees. Mycologia.

[b3] Agrios GN (1997). Plant Pathology.

[b4] Bacon CD, McKenna MJ, Simmons MP, Wagner WL (2012). Evaluating multiple criteria for species delimitation: an empirical example using Hawaiian palms (Arecaceae: *Pritchardia*. BMC Evol. Biol.

[b5] Bandelt HJ, Forster P, Rohl A (1999). Median-joining networks for inferring intraspecific phylogenies. Mol. Biol. Evol.

[b6] Bonito GM, Gryganskyi AP, Trappe JM, Vilgalys R (2010). A global meta-analysis of Tuber ITS rDNA sequences: species diversity, host associations and long-distance dispersal. Mol. Ecol.

[b7] Brasier CM, Kirk SA (2010). Rapid emergence of hybrids between the two subspecies of *Ophiostoma novo-ulmi* with a high level of pathogenic fitness. Plant. Pathol.

[b8] Brasier CM, Cooke DEL, Duncan JM (1999). Origin of a new *Phytophthora* pathogen through interspecific hybridization. Proc. Natl Acad. Sci. USA.

[b9] Carbone I, Anderson JB, Kohn LM (1999). A method for designing primer sets for speciation studies in filamentous Ascomycetes. Mycologia.

[b10] Chen C, Li M, Shi X, Wang J (1987). Studies on the infection period of *Valsa mali* Miyabe et Yamada, the causal agent of apple tree canker. Acta Phytopathologica Sinica.

[b11] Coyne JA, Orr HA (2004). Speciation.

[b12] Cummings MP, Neel MC, Shaw KL (2008). A genealogical approach to quantifying lineage divergence. Evolution.

[b13] Degnan JH, Rosenberg NA (2006). Discordance of species trees with their most likely gene trees. PLoS Genet.

[b14] Degnan JH, Rosenberg NA (2009). Gene tree discordance, phylogenetic inference and the multispecies coalescent. Trends Ecol. Evol.

[b15] Del-Prado R, Cubas P, Lumbsch TH, Divakar PK, Blanco O, de Paz GA (2010). Genetic distances within and among species in monophyletic lineages of Parmeliaceae (Ascomycota) as a tool for taxon delimitation. Mol. Phylogenet. Evol.

[b16] Dettman JR, Jacobson DJ, Taylor JW (2003). A multilocus genealogical approach to phylogenetic species recognition in the model eukaryote *Neurospora*. Evolution.

[b17] Dobzhansky T (1937). Genetics and the origin of species.

[b18] Drummond AJ, Rambaut A (2007). BEAST: Bayesian evolutionary analysis by sampling trees. BMC Evol. Biol.

[b19] Dupuis JR, Roe AD, Sperling FAH (2012). Multi-locus species delimitation in closely related animals and fungi: one marker is not enough. Mol. Ecol.

[b20] Fisher MC, Henk DA, Briggs CJ, Brownstein JS, Madoff LC, McCraw SL (2012). Emerging fungal threats to animal, plant and ecosystem health. Nature.

[b21] Gazis R, Rehner S, Chaverri P (2011). Species delimitation in fungal endophyte diversity studies and its implications in ecological and biogeographic inferences. Mol. Ecol.

[b22] Giraud T, Refrégier G, Hood M, Le Gac DM, de Vienne ME (2008). Speciation in fungi. Fungal Genet. Biol.

[b23] Giraud T, Gladieux P, Hood M (2010). The origin of species in Fungi. Fungi.

[b24] Glass NL, Donaldson GC (1995). Development of primer sets designed for use with the PCR to amplify conserved genes from filamentous Ascomycetes. Appl. Environ. Microbiol.

[b25] Guindon S, Gascuel O (2003). A simple, fast, and accurate algorithm to estimate large phylogenies by maximum likelihood. Systems biology.

[b26] Harder CB, LæssØe T, FrØslev TG, Ekelund F, Rosendahl S, KjØller R (2013). A three-gene phylogeny of the *Mycena pura* complex reveals 11 phylogenetic species and shows ITS to be unreliable for species identification. Fungal Biology.

[b27] Hausdorf B (2011). Progress toward a general species concept. Evolution.

[b28] Heled J, Drummond AJ (2010). Bayesian inference of species trees from multilocus data. Mol. Biol. Evol.

[b29] Hoffman MT, Arnold AE (2008). Geographic locality and host identity shape fungal endophyte communities in cupressaceous trees. Mycol. Res.

[b30] Huelsenbeck JP, Bull JJ, Cunningham CW (1996). Combining data in phylogenetic analysis. Trends Ecol. Evol.

[b31] Hull DL, Butts R, Hintikka J (1977). The ontological status of species as evolutionary units. Foundational problems in the special sciences.

[b32] Huson DH, Bryant D (2006). Application of phylogenetic networks in evolutionary studies. Mol. Biol. Evol.

[b33] Jargeat P, Martos F, Carriconde F, Gryta H, Moreau P-A, Gardes M (2010). Phylogenetic species delimitation in ectomycorrhizal fungi and implications for barcoding: the case of the *Tricholoma scalpturatum* complex (Basidiomycota). Mol. Ecol.

[b34] Jones KE, Patel NG, Levy MA, Storeygard A, Balk D, Gittleman JL (2008). Global trends in emerging infectious diseases. Nature.

[b35] Kasuga T, White TJ, Taylor JW (2002). Estimation of nucleotide substitution rates in Eurotiomycete fungi. Mol. Biol. Evol.

[b36] Lacap DC, Hyde KD, Liew ECY (2003). An evaluation of the fungal ‘morphotype’ concept based on ribosomal DNA sequences. Fungal Diversity.

[b37] Lê Van A, Gladieux P, Lemaire CornilleA, Giraud T, Durel C-E, Caffier V (2012). Evolution of pathogenicity traits in the apple scab fungal pathogen in response to the domestication of its host. Evol. Appl.

[b38] Leaché AD (2009). Species tree discordance traces to phylogeographic clade boundaries in North American fence lizards (*Sceloporus*. Systems biology.

[b39] Leavitt SD, Esslinger TL, Divakar PK, Lumbsch HT (2012). Miocene divergence, phenotypically cryptic lineages, and contrasting distribution patterns in common lichen-forming fungi (Ascomycota: Parmeliaceae). Biol. J. Linn. Soc. Lond.

[b40] Lee DH, Lee SW, Chi KH, Kim DA, Uhm JY (2006). Survey on the occurrence of apple disease in Korea from 1992 to 2000. Plant pathology journal.

[b41] Librado P, Rozas J (2009). DnaSP v5: A software for comprehensive analysis of DNA polymorphism data. Bioinformatics.

[b42] Lo WYY, Donoghue MJ (2012). Expanded phylogenetic and dating analyses of the apples and their relatives (Pyreae, Rosaceae). Mol. Phylogenet. Evol.

[b43] Lumbsch HT, Leavitt SD (2011). Goodbye morphology? A paradigm shift in the delimitation of species in lichenized fungi. Fungal Diversity.

[b44] Moon CD, Craven KD, Leuchtmann A, Clement SL, Schardl CL (2004). Prevalence of interspecific hybrids amongst asexual fungal endophytes of grasses. Mol. Ecol.

[b45] Newcombe G, Stirling B, McDonald S, Bradshaw HD (2000). *Melampsora* x *columbiana*, a natural hybrid of *M. medusae* and *M. occidentalis*. Mycol. Res.

[b46] Nilsson RH, Kristiansson E, Ryberg M, Hallbenberg N, Larsson K-H (2008). Intraspecific ITS variability in the kingdom Fungi as expressed in the international sequence databases and its implications for molecular species identification. Evolutionary bioinformatics.

[b47] Nylander JAA, Olsson U, Alström P, Sanmartín I (2008). Accounting for phylogenetic uncertainty in biogeography: a Bayesian approach to dispersal-vicariance analysis of the thrushes (Aves: Turdus). Systems biology.

[b48] O'Donnell K, Ward TJ, Geiser DM, Corby Kistler H, Aoki T (2004). Genealogical concordance between the mating type locus and seven other nuclear genes supports formal recognition of nine phylogenetically distinct species within the *Fusarium graminearum* clade. Fungal Genet. Biol.

[b49] Posada D, Crandall KA (1998). Modeltest: testing the model of DNA substitution. Bioinformatics.

[b50] Rambaut A, Drummond AJ (2007). http://beast.bio.ed.ac.uk/Tracer.

[b51] Rintoul T, Eggertson Q, Lévesque CA, Bolton M, Thomma BP (2012). Multigene phylogenetic analyses to delimit new species in fungal plant pathogens. Plant Fungal Pathogens: Methods and Protocols.

[b52] Ronquist F, Huelsenbeck JP (2003). MRBAYES 3: Bayesian phylogenetic inference under mixed models. Bioinformatics.

[b53] Rosenberg NA (2002). The probability of topological concordance of gene trees and species trees. Theor. Popul. Biol.

[b54] Schoch CL, Seifert KA, Huhndorf S, Robert V, Spouge JL, Levesque CA, Fungal Barcoding Consortium (2012). Nuclear ribosomal internal transcribed spacer (ITS) region as a universal DNA barcode marker for Fungi. Proc. Natl Acad. Sci. USA.

[b55] Sites JW, Crandall KA (1997). Testing species boundaries in biodiversity studies. Conserv. Biol.

[b56] Sterelny K, Wilson RA (1999). Species as ecological mosaics. Species: new interdisciplinary essays.

[b57] Stukenbrock EH, Christiansen FB, Hansen TT, Dutheil JY, Schierup MH (2012). Fusion of two divergent fungal individuals led to the recent emergence of a unique widespread pathogen species. Proc. Natl Acad. Sci. USA.

[b58] Takamatsu S, Matsuda S (2004). Estimation of molecular clocks for ITS and 28S rDNA in Erysiphales. Mycoscience.

[b59] Tamura O, Saito I (1982). Histopathological changes of apple bark infected by Valsa ceratosperma (Tode ex Fr.) Maire during dormant and growing periods. Nippon Shokubutsu Byori Gakkaiho.

[b60] Tamura K, Peterson D, Peterson N, Stecher G, Nei M, Kumar S (2011). MEGA5: molecular evolutionary genetics analysis using maximum likelihood, evolutionary distance, and maximum parsimony methods. Mol. Biol. Evol.

[b61] Taylor JW, Jacobson DJ, Kroken S, Kasuga T, Geiser DM, Hibbett DS (2000). Phylogenetic species recognition and species concepts in fungi. Fungal Genet. Biol.

[b62] Thompson JD, Gibson TJ, Plewniak F, Jeanmougin F, Higgins DG (1997). Clustal X windows interface: flexible strategies for multiple sequence alignment aided by quality analysis tools. Nucleic Acids Res.

[b63] Uhm JY, Sohn HR (1995). Control of apple Valsa canker by localized spraying with neoasozin solution, an arsenic fungicide. Korean Journal of Plant Resources.

[b64] Verkley GJM, Quaedvlieg W, Shin H-D, Crous PW (2013). A new approach to species delimitation in *Septoria*. Stud. Mycol.

[b65] Wang L, Zang R, Huang LL, Xie FQ, Gao XN (2005). The investigation of apple tree Valsa canker in Guanzhong region of Shaanxi province. Journal of Northwest Sci-Tech University of Agriculture and Forestry.

[b66] Wang XL, Kang ZS, Huang LL, Yang P (2007). Pathogen identification of Valsa canker on pear tree: evidences from rDNA-ITS sequences and cultural characteristics. Mycosystema.

[b67] Wang XL, Wei JL, Huang LL, Kang ZS (2011). Re-evaluation of pathogens causing Valsa canker on apple in China. Mycologia.

[b68] White TJ, Bruns TD, Lee SB, Taylor JW, Innis MA, Gelfand DH, Sninsky JJ, White TJ (1990). Amplification and direct sequencing of fungal ribosomal RNA genes for phylogenetics. PCR protocols, a guide to methods and applications.

[b69] Yang Z, Rannala B (2010). Bayesian species delimitation using multilocus sequence data. Proc. Natl Acad. Sci. USA.

[b70] Yu Y, Harris AJ, He X (2010). S-DIVA (Statistical Dispersal-Vicariance Analysis): a tool for inferring biogeographic histories. Mol. Phylogenet. Evol.

[b71] Zang R, Ke XW, Wang XJ, Li ZL, Kang ZS, Huang LL (2012). A nested PCR assay for detecting *Valsa mali* var. *mali* in different tissues of apple trees. Plant Dis.

