# Supplementary material for: Delimiting cryptic pathogen species causing apple Valsa canker with multilocus data
Source: Ecol Evol. 2014 Mar 19;4(8):1369–80. doi: 10.1002/ece3.1030 (PMC4020696; doi:10.1002/ece3.1030)
Supplement: Supplementary file 1 — Table S1. Isolates, host origins, and GenBank accession numbers of isolates of Valsa species. Sequences for VleucostomaFLN32b were obtained from GenBank. Table S2. Results of likelihood ratio test of molecular clock in three loci (ITS, Btu, and EF1α). [file ece30004-1369-sd1.docx]

Table S1. Isolates, host origins and GenBank accession numbers of isolates of *Valsa* species. Sequences for VleucostomaFLN32b was obtained from Genbank.

| **Isolates** | **Species** | **Hosts** | **ITS** | **EF1α** | **Btu** |
| --- | --- | --- | --- | --- | --- |
| Vm008 | *V. mali* | *Malus* sp. | JN412599 | JQ900314 | JQ900344 |
| Vm024 | *V. mali* | *Malus* sp. | GU174588 | JQ900313 | JQ900348 |
| Vm027 | *V. mali* | *Malus* sp. | JN412608 | JQ900322 | JQ900356 |
| Vm054 | *V. mali* | *Malus* sp. | JN792569 | JQ900316 | JQ900342 |
| Vm069 | *V. mali* | *Malus* sp. | JN412602 | JQ900317 | JQ900347 |
| Vm092 | *V. mali* | *Malus* sp. | JN412604 | JQ900312 | JQ900354 |
| Vm109 | *V. mali* | *Malus* sp. | GU174584 | JQ900323 | JQ900357 |
| Vm118 | *V. mali* | *Malus* sp. | JN412605 | JQ900321 | JQ900353 |
| Vm119 | *V. mali* | *Malus* sp. | GU174581 | JQ900320 | JQ900353 |
| Vm123 | *V. mali* | *Malus* sp. | JN792573 | JQ900311 | JQ900355 |
| Vm142 | *V. mali* | *Malus* sp. | JN792572 | JQ900309 | JQ900349 |
| Vm143 | *V. mali* | *Malus* sp. | JN792571 | JQ900310 | JQ900350 |
| Vm146 | *V. mali* | *Malus* sp. | GU174587 | JQ900308 | JQ900343 |
| Vm152 | *V. mali* | *Malus* sp. | GU174585 | JQ900319 | JQ900351 |
| Vm156 | *V. mali* | *Malus* sp. | JN792570 | JQ900315 | JQ900345 |
| Vm169 | *V. mali* | *Malus* sp. | JN792574 | JQ900318 | JQ900346 |
| Vp014 | *V. pyri* | *Malus* sp. | JN673554 | JQ900327 | JQ900362 |
| Vp126 | *V. pyri* | *Malus* sp. | JN662365 | JQ900326 | JQ900361 |
| Vp134 | *V. pyri* | *Malus* sp. | JN662366 | JQ900325 | JQ900359 |
| Vp135 | *V. pyri* | *Malus* sp. | JN662367 | JQ900330 | JQ900358 |
| Vp157 | *V. pyri* | *Pyrus* sp*.* | JN662369 | JQ900332 | JQ900366 |
| Vp160 | *V. pyri* | *Pyrus* sp*.* | JN662370 | JQ900328 | JQ900365 |
| Vp161 | *V. pyri* | *Pyrus* sp | JN662371 | JQ900331 | JQ900364 |
| Vp162 | *V. pyri* | *Pyrus* sp | JN662372 | JQ900329 | JQ900360 |
| Vp165 | *V. pyri* | *Pyrus* sp | JN792568 | JQ900324 | JQ900363 |
| Vmalicola001 | *V. malicola* | *Malus* sp. | JN545839 | JQ900336 | JQ900371 |
| Vmalicola007 | *V. malicola* | *Malus* sp. | JN792575 | JQ900337 | JQ900370 |
| Vmalicola136 | *V. malicola* | *Malus* sp. | GU174579 | JQ900335 | JQ900368 |
| Vmalicola137 | *V. malicola* | *Malus* sp. | GU174578 | JQ900334 | JQ900369 |
| Vmalicola138 | *V. malicola* | *Malus* sp. | JN792576 | JQ900333 | JQ900367 |
| Vleucostoma32w | *V. leucostoma* | *Malus* sp. | JN584644 | JQ900340 | JQ900374 |
| VleucostomaTF | *V. leucostoma* | *Prunus* sp. | JN792579 | JQ900339 | JQ900373 |
| VleucostomaFLN32b | *V. leucostoma* | *Pyrus communis* | KF293297 | KF293296 | KF293298 |

Table S2. Results of likelihood ratio test of molecular clock in three loci (ITS, Btu and EF1α).

| Locus | λ_non-clock_ | λ_clock_ | 2Δ | d.f. | *P* |
| --- | --- | --- | --- | --- | --- |
| ITS | -959.14 | -1046.04 | 173.80 | 28 | <0.0001 |
| Btu | -1171.47 | -1342.49 | 342.04 | 28 | <0.0001 |
| EF1α | -840.95 | -1037.16 | 392.42 | 28 | <0.0001 |
| Concatenated | -3192.38 | -3584.01 | 783.26 | 28 | <0.0001 |
