# Supplementary figures and images for: Delimiting cryptic pathogen species causing apple Valsa canker with multilocus data
Source: Ecol Evol. 2014 Mar 19;4(8):1369–80. doi: 10.1002/ece3.1030 (PMC4020696; doi:10.1002/ece3.1030)

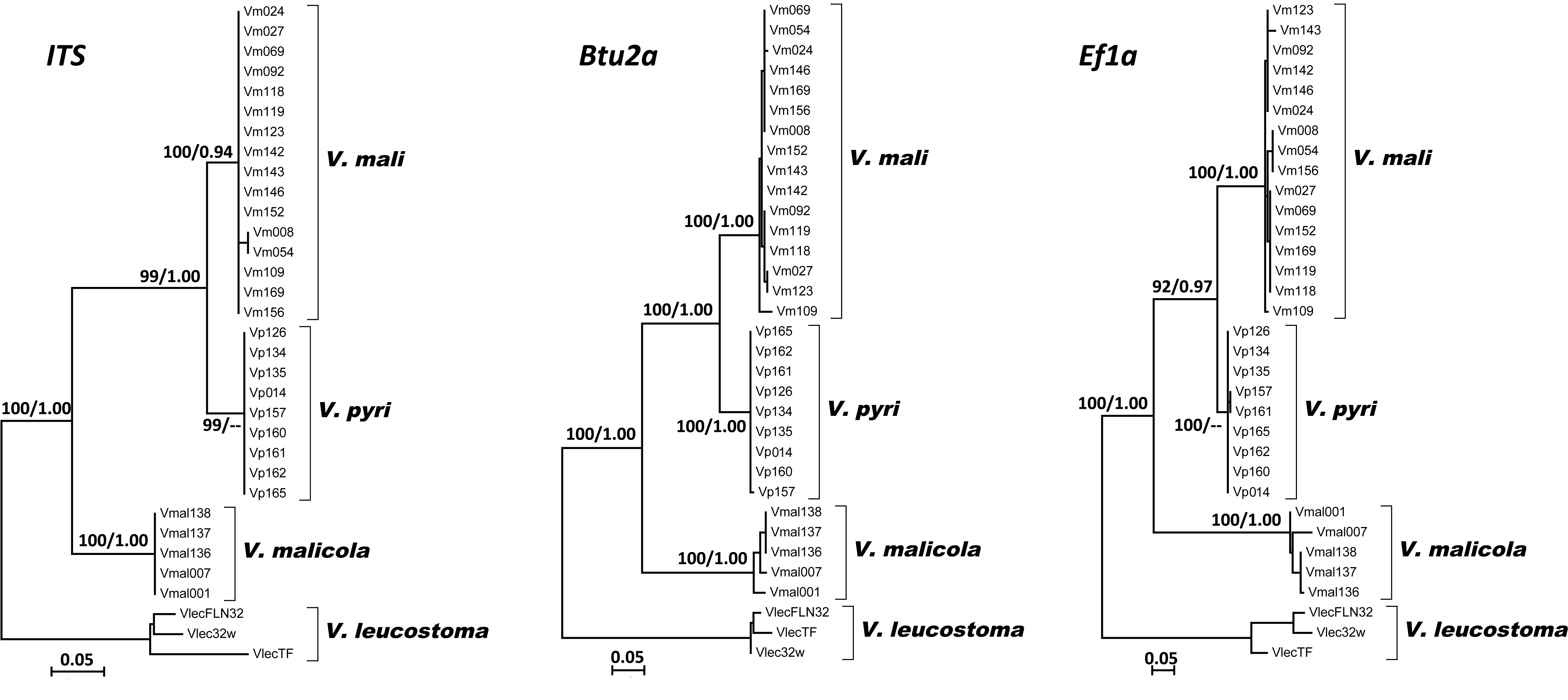

Supplement: Figure S1 — Maximum likelihood tree inferred from ITS (left), Btu (middle), and EF1α (right). Bootstrapping values are shown together with Bayesian posterior probabilities at the node. [file ece30004-1369-sd2.jpg]
